# Clinical Frailty Scale (CFS) reliably stratifies octogenarians in German ICUs: a multicentre prospective cohort study

**DOI:** 10.1186/s12877-018-0847-7

**Published:** 2018-07-13

**Authors:** Johanna M. Muessig, Amir M. Nia, Maryna Masyuk, Alexander Lauten, Anne Lena Sacher, Thorsten Brenner, Marcus Franz, Frank Bloos, Henning Ebelt, Stefan J. Schaller, Kristina Fuest, Christian Rabe, Thorben Dieck, Stephan Steiner, Tobias Graf, Rolf A. Jánosi, Patrick Meybohm, Philipp Simon, Stefan Utzolino, Tim Rahmel, Eberhard Barth, Michael Schuster, Malte Kelm, Christian Jung

**Affiliations:** 1 Division of Cardiology, Pulmonology, and Vascular Medicine, University Hospital Düsseldorf, Moorenstr. 5, 40225 Düsseldorf, Germany; 2 CARID, Cardiovascular Research Institute Düsseldorf, Düsseldorf, Germany; 3 Department of Cardiology, Charité - University Hospital, Berlin, Germany; 4 German Center for Heart Research (DZHK), Berlin, Germany; 5 Department of Anaesthesiology and Operative Intensive Care Medicine, Campus Charité Mitte and Campus Virchow-Klinikum, Berlin, Germany; 6 Department of Anaesthesiology, Heidelberg University Hospital, Heidelberg, Germany; 7 Department of Internal Medicine I, Friedrich-Schiller-University, University Hospital Jena, Jena, Germany; 8 Department of Anaesthesiology and Intensive Care Medicine, University Hospital Jena, Jena, Germany; 9 Department for Medicine II, Catholic Hospital “St. Johann Nepomuk”, Erfurt, Germany; 10 Department of Anaesthesiology, Klinikum rechts der Isar, Technical University Munich, Munich, Germany; 11 Department of Clinical Toxicology, Klinikum rechts der Isar, Technical University Munich, Munich, Germany; 12 Department of Anaesthesiology and Intensive Care, Hannover Medical School, Hannover, Germany; 13 St. Vincenz Hospital, Department of Cardiology, Pneumology and Intensive Care Medicine, Limburg/Lahn, Limburg, Germany; 14 University Heart Center Luebeck, Department of Cardiology, Angiology, and Intensive Care Medicine, University Hospital Schleswig-Holstein, Luebeck, Germany; 15 German Center for Cardiovascular Research (DZHK), Partner Site Hamburg/Kiel/Luebeck, Luebeck, Germany; 16 Medical Faculty, West German Heart and Vascular Center, Department of Cardiology and Vascular Diseases, University Hospital Essen, Essen, Germany; 17 Department of Anaesthesiology, Intensive Care Medicine and Pain Therapy, University Hospital Frankfurt, Frankfurt/Main, Germany; 18 Department of Anaesthesiology and Intensive Care Medicine, University Hospital of Leipzig, Leipzig, Germany; 19 Department of General and Visceral Surgery, University Hospital Freiburg, Freiburg, Germany; 20 Department of Anaesthesiology, Intensive Care Medicine and Pain Therapy, University Hospital Knappschaftskrankenhaus Bochum, Bochum, Germany; 21 Division of Intensive Care Medicine, Department of Anaesthesiology, University Hospital Ulm, Ulm, Germany; 22 Department of Anaesthesiology, Intensive Care Medicine and Pain Therapy, University Hospital Mainz, Mainz, Germany

**Keywords:** Frailty, Clinical frailty scale, VIP1, Intensive care outcome

## Abstract

**Background:**

In intensive care units (ICU) octogenarians become a routine patients group with aggravated therapeutic and diagnostic decision-making. Due to increased mortality and a reduced quality of life in this high-risk population, medical decision-making a fortiori requires an optimum of risk stratification. Recently, the VIP-1 trial prospectively observed that the clinical frailty scale (CFS) performed well in ICU patients in overall-survival and short-term outcome prediction. However, it is known that healthcare systems differ in the 21 countries contributing to the VIP-1 trial. Hence, our main focus was to investigate whether the CFS is usable for risk stratification in octogenarians admitted to diversified and high tech German ICUs.

**Methods:**

This multicentre prospective cohort study analyses very old patients admitted to 20 German ICUs as a sub-analysis of the VIP-1 trial. Three hundred and eight patients of 80 years of age or older admitted consecutively to participating ICUs. CFS, cause of admission, APACHE II, SAPS II and SOFA scores, use of ICU resources and ICU- and 30-day mortality were recorded. Multivariate logistic regression analysis was used to identify factors associated with 30-day mortality.

**Results:**

Patients had a median age of 84 [IQR 82–87] years and a mean CFS of 4.75 (± 1.6 standard-deviation) points. More than half of the patients (53.6%) were classified as frail (CFS ≥ 5). ICU-mortality was 17.3% and 30-day mortality was 31.2%. The cause of admission (planned vs. unplanned), (OR 5.74) and the CFS (OR 1.44 per point increase) were independent predictors of 30-day survival.

**Conclusions:**

The CFS is an easy determinable valuable tool for prediction of 30-day ICU survival in octogenarians, thus, it may facilitate decision-making for intensive care givers in Germany.

**Trial registration:**

The VIP-1 study was retrospectively registered on ClinicalTrials.gov (ID: NCT03134807) on May 1, 2017.

**Electronic supplementary material:**

The online version of this article (10.1186/s12877-018-0847-7) contains supplementary material, which is available to authorized users.

## Background

Very old patients (age ≥ 80 years) now comprise approximately one fourth of all intensive care unit (ICU) admissions in the United States and similarly in Europe, receive much more intensive medical treatment than in the past, and progressively survive even fatal critical illnesses [1, 2]. However, in view of enormous personal efforts of intensive care givers and increased economic costs due to prolonged and complicated ICU stays of octogenarians, proportionality of costs and resource usage came in focus [2] with two main aspects: First, emergency triage process must be optimised and simplified to facilitate ICU admission for very old patients [3, 4]. Second, more scientific and clinical interest must be applied on all aspects of geriatric medicine to adapt existing risk-stratification data to daily clinical scenarios [5, 6].

In this context, a 9-point Clinical Frailty Scale (CFS) as an advancement of the Canadian 7-point CFS has been developed [7]. Recently, the prospective, observational, European-ICU-network based VIP-1 study demonstrated that frailty is a significant factor reducing 30-day survival and that the 9-point CFS is an appropriate tool to assess this effect [4]. The rationale behind scientific efforts in this field is explained by the very complex nature of circumscribing frailty to other specific medical problems [8]. Consecutively, whereas physical frailty is a measurable clinical phenotype of increased vulnerability for developing adverse outcomes (e.g. disability and/or mortality) when exposed to a stressor, it is highly difficult to explain the current frailty status in an abbreviated and objective manner to another medical-professional.

As the largest study investigating the value of preadmission CFS in elderlies admitted to ICUs, the VIP-1 study was one of the first large studies highlighting the impact of assessing CFS as a herald for outcome in very old ICU patients [4]. The advanced 9-point CFS, measured at a rapid diagnostic glance, showed even more robust outcome-foreshowing value compared to routinely used ICU indices like SOFA or APACHE-II score in VIP-1 [4].

ICUs from 21 European countries and 3 Non-Europe countries participated in the VIP-1 study [4]. The participating countries represent a broad range of different health care systems and philosophies, and less than 10% of included patients came from Germany. The recent Organisation for Economic Co-operation and Development (OECD) health report emphasized the differences in health status and life expectancy in the participating countries, leading to varying challenges [9]. These factors most likely affect case mixes and intensity of treatment provided in ICUs of the different countries participating in the VIP-1 trial. Indeed, VIP-1 showed regional differences in the frequency of frail patients admitted to ICUs. Consequently, the conclusions drawn by the multi-national VIP-1 study might not be transferable to the individual health care situation of each participating country. In this current study, we undertook a multicentre sub-analysis of the German VIP-1 cohort and aimed to test if the predictive value of CFS is still robust on a national level in very diversified and high-tech German ICUs.

## Methods

### Study sample

**Fig. 1:**
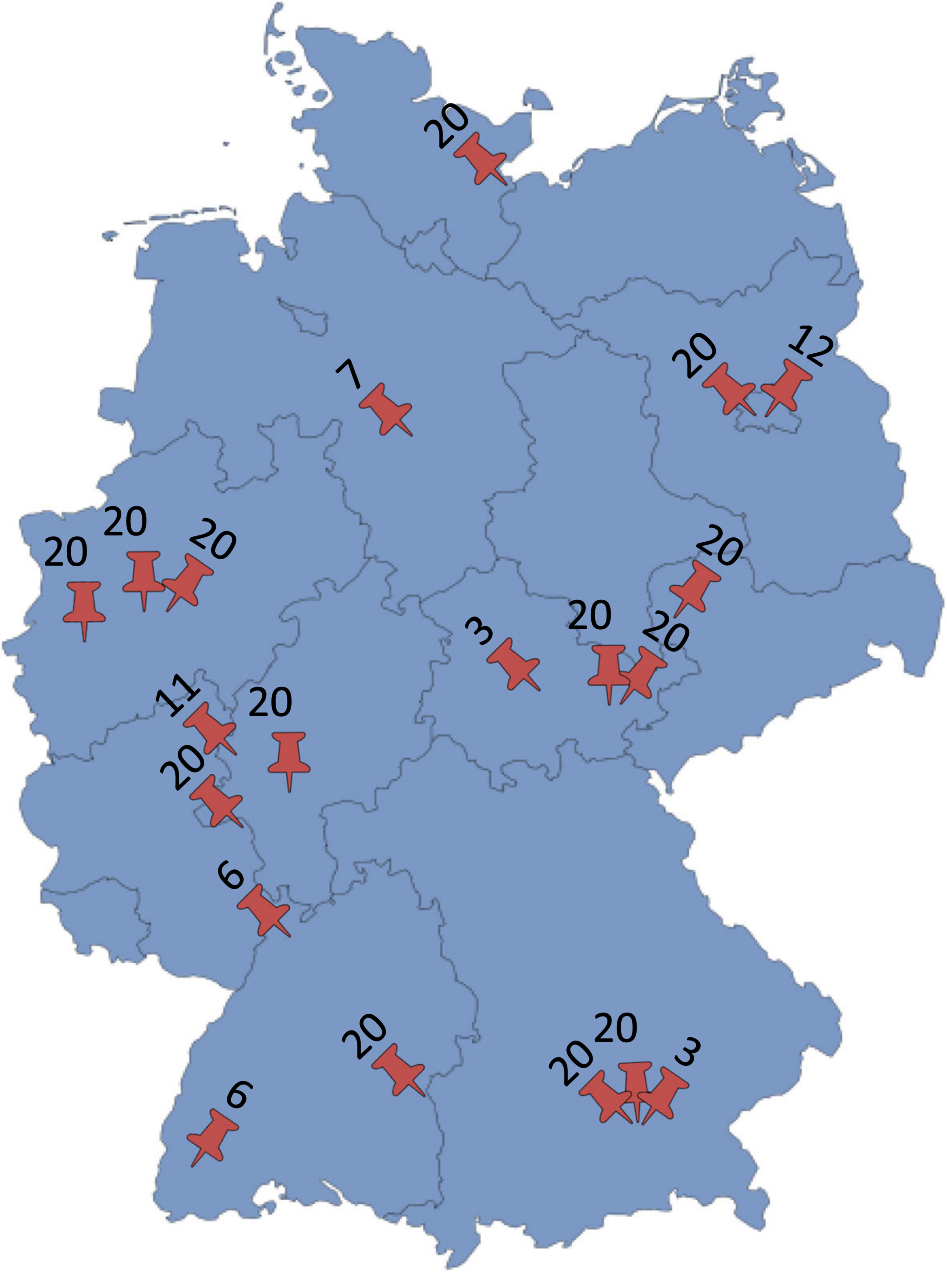
Participating ICUs. Participating ICUs are marked. The number of enclosed patients is indicated for each participating ICU

This analysis is a sub-analysis of the 20 German ICUs participating in the prospectively realised observational multicentre European VIP-1 study (Fig. 1) [4]. The aim of the study was to investigate whether the CFS is usable for risk stratification in octogenarians admitted to diversified and high tech German ICUs. The VIP-1 study was coordinated by the Health Services Resource and Outcome (HSRO) section of the European Society of Intensive Care Medicine (ESICM) and was registered on ClinicalTrials.gov (ID: NCT03134807; first posted on May 1st 2017 after recruitment completed). All patients at least 80 years of age admitted to the participating ICUs for any cause were eligible for documentation after giving written informed consent (by themselves or responsible advisor). Data from not more than 20 consecutive patients per ICU were collected.

Ethical approval was obtained from each study side (Additional file 1: Ethic committees). Patients were recruited between October 2016 and February 2017.

### Data collection and scoring systems

Initial Simplified Acute Physiology Score II (SAPS II), Acute Physiology And Chronic Health Evaluation II (APACHE II) and Sequential Organ Failure Assessment (SOFA) scores were calculated by treating physicians within 24 h after admission as reported previously [10–12]. Length of stay (LOS) was recorded in hours, allowing calculation of LOS in 24-h periods rather than in calendar days. It was recorded whether patients were subjected to invasive or non-invasive ventilation, vasoactive drugs were administered or renal replacement therapy was applied during their ICU stay. However, the duration of those intensive therapies was not recorded. The database was located on a secure server in Aarhus University, Denmark.

### Frailty

Frailty was defined according to the Clinical Frailty Scale (CFS). The CFS is an easy and intuitive determinable categorisation tool based on simple visual descriptions [7]. The CFS divides patients into 9 classes from very fit (CFS = 1) to terminally ill (CFS = 9). The status of the patients before hospital admission was obtained from the patients or their relatives.

### Statistical analyses

Statistical analyses were performed using SPSS (IBM Corp. Released 2013. IBM SPSS Statistics for Windows, Version 22.0. Armonk, NY, USA). For statistical analyses, patients were stratified according to their admission status (planned vs. unplanned). The patients with unplanned admission were further divided into the following three groups: acute medical, acute surgery and trauma. Patients were followed for 30 days after ICU admission. Baseline patient characteristics, treatment and outcomes were compared between two frailty groups based on the CFS: not-frail (CFS 1–4) and frail (CFS 5–9) as used in previous publications [6].

Additionally, for characterisation of patients according to 30-day survival and according to the type of admission, frailty status was defined as not frail (CFS 1–3), pre-frail (CFS 4) and frail (CFS 5–9) [4]. Frailty was also analysed using the whole scale as ordinal data. For continuous variables, normally distributed data is given in mean ± standard deviation and compared by student’s t-test. Non-normally distributed data is shown as median with inter-quartile-range and compared by Mann-Whitney-U-Test or Kruskal-Wallis-Test as appropriate. Categorical variables were described by counts and percentages. Differences between groups were calculated by Chi-square test. The impact of age, gender, APACHE II, SAPS II, Sofa-score, type of admission (planned vs. unplanned, acute medical vs. acute surgery, acute surgery vs. trauma and acute medical vs. trauma) and frailty on 30-day mortality was estimated using logistic regression analyses. Variables with a *p*-value below 0.10 in univariate logistic regression analysis were included in the multivariate logistic regression model to estimate the adjusted impact of frailty on 30-day mortality. *P*-values < 0.05 were considered significant.

## Results

In this sub-analysis of the European VIP-1 study cohort, a total number of 308 patients of 80 years of age or older admitted to 20 German ICUs were analysed. Participating ICUs collected data from 3 to 20 patients (Fig. 1).

**Fig. 2:**
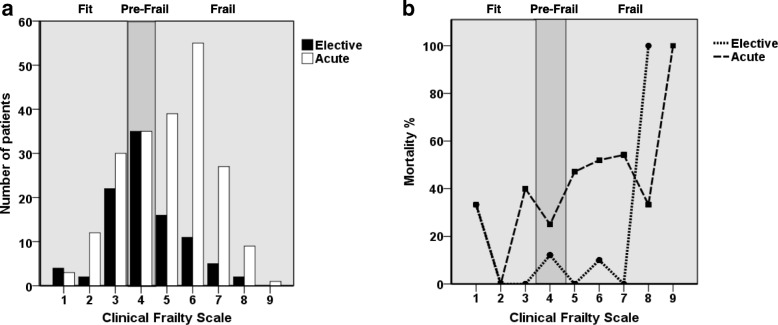
Association of the Clinical Frailty Scale with 30-day mortality in acute admitted patients. Distribution of frailty in acute and elective admitted Patients (**a**). Association of frailty with 30-day mortality (**b**). Patients were divided into three groups according to their frailty status: fit (CFS 1–3), pre-frail (CFS 4) and frail (CFS 5–9). CFS: Clinical Frailty Score ranging from 1 (not frail) to 9 (terminally ill)

Median age was 84 [IQR 82–87] years and 50% of the patients were male. Median LOS in the ICU was 3.1 [IQR 1.1–8.1] days. ICU-mortality was 17.3% and 30-day mortality was 31.2% (Table 1). Out of the analysed ICU patients, 31.5% had planned admissions due to post surgery care. The other patients were admitted due to acute medical reasons (52.6%), trauma (10.7%) or acute surgery (5.2%) as shown in Table 1 and Fig. 2. Respiratory and/or circulatory failure was the most frequent cause for admission. Use of life sustaining treatments is shown in Table 1. In 26.6% of the patients none of the ICU procedures were recorded. Frailty according to the CFS could be determined for all patients.

**Table 1:** Patient characteristics according to the frailty status

Frailty status	Overall	Not frail (CFS 1–4)	Frail (CFS 5–9)	*p*-value
Number of admissions	308 (100%)	143 (46.4%)	165 (53.6%)	
Age	84 [82–87]	82 [81–86]	85 [82–88]	< 0.001
Male	164 (50%)	79 (55.2%)	75 (45.5%)	0.09
APACHE II	18 [12–26]	15 [12–25]	19 [12–26]	0.18
SAPS II	39 [29.5–51]	37 [29–48]	42 [30–55]	0.17
SOFA Score	6 [3–10]	6 [3–9]	7 [4–10]	0.016
Cause of admission				
Acute medical	162 (52.6%)	58 (40.6%)	104 (63%)	< 0.001
Acute surgery	16 (5.2%)	8 (5.6%)	8 (4.8%)	0.77
Trauma	33 (10.7%)	14 (9.8%)	19 (11.5%)	0.63
Elective surgery	97 (31.5%)	63 (44.1%)	34 (20.6%)	< 0.001
ICU length of stay (days)	3.1 [1.1–8.1]	2.9 [1–7]	3.4 [1.4–9.5]	0.21
Use of life sustaining treatments				
Non-invasive ventilation	104 (33.8%)	48 (33.6%)	56 (33.9%)	0.95
Mechanical ventilation	141 (45.8%)	59 (41.3%)	82 (49.7%)	0.14
Vasoactive drugs	189 (61.4%)	82 (57.3%)	107 (64.8%)	0.18
Renal replacement therapy	59 (19.2%)	26 (18.2%)	33 (20%)	0.69
Treatment withheld	41 (13.3%)	11 (7.7%)	30 (18.2%)	0.01
Treatment withdrawn	36 (11.7%)	10 (7%)	26 (15.8%)	0.02
ICU mortality	53 (17.3%)	16 (11.3%)	37 (22.4%)	0.01
30-day mortality	84 (31.2%)	23 (18.4%)	61 (42.4%)	< 0.001

Frail patients are characterized by frailty score ≥ 5 and not frail patients by a frailty score ≤ 4. Data are presented as median and interquartile range [IQR] or frequency and percentages (n, %). ICU: Intensive Care Unit. CFS: Clinical Frailty Score ranging from 1 (not frail) to 9 (terminally ill).

Planned admitted patients had a lower CFS compared to acute admitted patients and showed a lower ICU- and 30-day mortality (Fig. 2, Table 2). Data are presented as median and interquartile range [IQR] or frequency and percentages (n, %). ICU: Intensive Care Unit. CFS: Clinical Frailty Score ranging from 1 (not frail) to 9 (terminally ill).

**Table 2:** Patient characteristics according to the cause of admission

Admission unplannd vs. planned		Unplanned		Planned	*p-*value
Cause of admission	Acute medical	Acute surgery	Trauma	Elective surgery	Elective vs acute
Sample size	162 (52.6%)	16 (5.2%)	33 (10.7%)	97 (31.5%)	
Age	84 [82–86]	83 [81–89]	86 [83–90]	83 [81–87]	0.33
Male	82 (50.6%)	12 (75%)	13 (39.4%)	47 (48.5%)	0.71
APACHE II	20 [15–27]	18 [14–18]	18 [13–24]	14 [10–19]	0.001
SAPS II	42 [34–53]	46 [42–46]	31 [23–46]	35 [26–49]	0.03
SOFA Score	8 [4–10]	11 [6–13]	6 [4–9]	4 [3–7]	< 0.001
Frailty status					
Fit (CFS 1–3)	30 (18.5%)	7 (43.8%)	8 (24.2%)	28 (28.9%)	0.15
Pre-frail (CFS 4)	28 (17.3%)	1 (6.3%)	6 (18.2%)	35 (36.1%)	< 0.001
Frail (CFS 5–9)	104 (64.2%)	8 (50%)	19 (57.6%)	34 (35.1%)	< 0.001
ICU length of stay (days)	5 [2.0–10.5]	6.2 [2.1–17.6]	3 [1.3–6.8]	1.4 [1–3.5]	< 0.001
Use of life sustaining treatments					
Non-invasive ventilation	63 (38.9%)	6 (37.5%)	10 (30.3%)	25 (25.8%)	0.04
Mechanical ventilation	75 (46.3%)	13 (81.3%)	20 (60.6%)	33 (34%)	0.005
Vasoactive drugs	110 (67.9%)	15 (93.8%)	20 (60.6%)	44 (45.4%)	< 0.001
Renal replacement therapy	41 (25.3%)	4 (25%)	1 (3%)	13 (13.4%)	0.08
Treatment withheld	29 (17.9%)	3 (18.8%)	9 (27.3%)	0 (0%)	< 0.001
Treatment withdrawn	27 (16.7%)	3 (18.8%)	4 (12.1%)	2 (2.1%)	< 0.001
ICU mortality	39 (24.1%)	4 (25%)	6 (18.2%)	4 (4.2%)	< 0.001
30-day mortality	61 (41.8%)	6 (50%)	10 (38.5%)	7 (8.2%)	< 0.001

For further evaluation of the impact of frailty, patients were divided into two groups according to their frailty status as depicted in Table 1. Frail patients showed higher values for organ failure and ICU treatment was more often withhold or withdrawn. However, there was no significant difference between frail and non-frail patients with regard to the APACHE-II and SAPS-II scores. ICU mortality and 30-day mortality were significantly higher for frail patients compared to non-frail patients (Table 1 and Fig. 2). Patients surviving 30 days after ICU admission were less frail, stayed shorter in ICU, got less invasive treatment and treatment was less often withheld or withdrawn (Table 3). Data are presented as interquartile range [IQR] or absolute numbers and percentages (n, %). ICU: Intensive Care Unit. CFS: Clinical Frailty Score ranging from 1 (not frail) to 9 (terminally ill).

**Table 3:** Patient characteristics according to the survival status

30 day survival status	Survivors	Nonsurvivors	*p* value
Number of admissions	185 (68.8%)	84 (31.2%)	
Age	83 [81–86]	85 [81–88]	0.06
Male	85 (45.9%)	47 (56%)	0.13
APACHE II	17 [12–23]	25 [18–32]	< 0.001
SAPS II	37 [29–50]	48 [39–61]	0.001
SOFA Score	6 [3–9]	8 [5–11]	< 0.001
Frailty status			
Fit (CFS 1–3)	52 (28.1%)	12 (14.3%)	0.014
Pre-frail (CFS 4)	50 (27%)	11 (13%)	0.011
Frail (CFS 5–9)	83 (44.9%)	61 (72.6%)	< 0.001
Cause of admission			
Acute medical	85 (45.9%)	61 (72.6%)	< 0.001
Acute surgery	6 (3.2%)	6 (7.1%)	0.15
Trauma	16 (8.6%)	10 (11.9%)	0.4
Elective surgery	78 (42.2%)	7 (8.3%)	< 0.001
ICU length of stay (days)	2.7 [1–8]	5.2 [2.2–9.8]	0.012
Use of life sustaining treatments			
Non-invasive Ventilation	69 (37.3%)	28 (33.3%)	0.53
Mechanical ventilation	74 (40%)	58 (69%)	< 0.001
Vasoactive drugs	107 (57.8%)	66 (78.6%)	0.001
Renal replacement therapy	31 (16.8%)	27 (32.1%)	0.004
Treatment withheld	8 (4.3%)	32 (38.1%)	< 0.001
Treatment withdrawn	1 (0.5%)	34 (40.5%)	< 0.001
ICU mortality	0 (0%)	53 (63.1%)	< 0.001

Univariate regression analysis showed that frailty measured by the CFS as well as APACHE II, SAPS II and SOFA scores and the cause of admission (unplanned vs. planned) were associated with higher 30-day mortality. In a multivariate regression analysis the cause of admission was the strongest predictor for 30-day mortality (OR 5.74) after adjusting for other covariates. Thus, mortality was 5.74 times higher in acute admitted patients compared to elective admitted patients. Moreover, the CFS independently predicted 30-day mortality in the multivariate regression analyses (OR 1.44). Thus, for each point increase of the CFS, mortality increased by 1.4 times. However, the APACHE II, SAPS II and SOFA scores failed to predict 30-day mortality in a multivariate regression analysis (Table 4).

**Table 4:** Survival analyses: Univariate and multivariate logistic regression analyses

Parameter	Univariate predictor			Multivariable predictor model		
	OR	95% CI	*p* value	AOR	95% CI	*p* value
Age (per year increase)	1.069	[0.999;1.114]	0.053	1.040	[0.920; 1.177]	0.531
Male	0.669	[0.398; 1.124]	0.13			
APACHE II (per point increase)	1.101	[1.046; 1.158]	<0.001	1.053	[0.982; 1.129]	0.149
SAPS II (per point increase)	1.024	[1.006; 1.043]	0.01	1.007	[0.973; 1.043]	0.672
SOFA Score (per point increase)	1.150	[1.075; 1.231]	<0.001	1.072	[0.914; 1.259]	0.392
Type of admission						
Acute vs. elective	8.019	[3.507; 18.333]	<0.001	5.744	[1.186; 27.811]	0.030
Acute medical vs acute surgery	0.718	[0.221; 2.332]	0.581			
Acute surgery vs. trauma	1.600	[0.402; 6.361]	0.504			
Acute medical vs. trauma	1.148	[0.488; 2.702]	0.752			
Frailty						
Frailty (per point increase)	1.410	[1.187; 1.675]	<0.001	1.437	[1.052; 1.964]	0.023
Frail (CFS 5-9)	3.259	[1.861; 5.708]	<0.001			
Pre-frail (CFS 4)	0.407	[0.200; 0.829]	0.013			
Fit (CFS 1-3)	0.426	[0.214; 0.850]	0.015			

Impact of the different factors on 30-day mortality in a univariate model is presented in the left part of the table. Factors with a *p*-value below 0.10 in a univariate logistic regression analysis were included into the multivariate model shown in the right part of the table. Impact of the factors on 30-day mortality is presented as Odd’s Ratio (OR) in the univariate model or as adjusted Odd’s Ratio (AOR) in the multivariate model respectively, and 95% Confidence Interval (95% CI). CFS: Clinical Frailty Score ranging from 1 (not frail) to 9 (terminally ill).

## Discussion

This multicentre analysis of the German VIP-1 cohort [4] focussing on a large cohort of more than 300 very old patients admitted to 20 German ICUs showed that frailty measured by the 9-point CFS [4] is a valuable predictor of ICU- and 30-day mortality for very old patients admitted to highly diversified and high-tech German ICUs. These findings complement the recent analyses of the multi-national, prospective European VIP-1 cohort [4]. In previous publications, conducted mainly in single centres, huge differences of ICU survival rates among octogenarians ranging from 14 to 46% have been described [13, 14]. This divergence can most likely be explained by case mix differences. Thus, the multi-national European VIP-1 study showed that in central, northern and eastern Europe a higher percentage of the admitted patients were frailer than in southern and western European countries [4]. This finding is also reflected by the recent OECD health report pointing out differences in health care systems in the OECD countries [9]. Our analysis of very old patients in German ICUs showed an ICU mortality of 17.3% and a 30-day mortality of 31.2% similar to the multi-national VIP-1 cohort with 22.1 and 32.4% and to a Canadian cohort with 21.8 and 35% [15], respectively. However, despite high mortality rates, it is difficult to identify patients at risk among that vulnerable cohort since commonly used scoring systems for prediction of ICU outcome were developed for younger cohorts and are not reliable for use in the very old [16]. Thus, in contrast to the results of the multi-national VIP-1 cohort, APACHE II, SAPS II and the SOFA score failed to predict 30-day mortality in a multivariate regression analysis on our multicentre cohort of very old patients in German ICUs. Recently, it was suggested that frailty is correlated with mortality in very old patients and that quantification of frailty might predict the outcome of very old patients admitted to ICUs. Frailty is defined as a condition of clinical vulnerability associated with age dependent reduction of physiological reserves [17]. Different scores for the assessment of frailty were developed and their ability for the prediction of adverse outcomes including decline in functional performance, prolonged length of stay, institutionalisation, and mortality in geriatric patient collectives was confirmed [4, 18–22]. Using the previously reported 9-point CFS [4], we could show that increased frailty was independently associated with intra-ICU and 30-day mortality in a multicentre cohort of very old patients admitted to German ICUs. Thus, our data confirmed the observations of previous studies from Europe [4] and America [23] and proofed the usability of the CFS for risk stratification of octogenarians in German ICUs. The only parameter that was stronger associated with 30-day mortality than the frailty status was the type of admission (planned vs. unplanned). That observation might be caused by a selection bias since our analyses revealed that electively admitted patients were less frail and showed lower APACHE II, SAPS II and SOFA scores. Furthermore, health status of patients selected for elective surgery could be optimized prior to ICU admission. In contrast to the analysis of the multi-national European VIP-1 cohort [4], our sub-analysis of the German VIP-1 cohort revealed no independent impact of age, gender or the SOFA score on 30-day mortality in a multivariate regression analysis. This important difference between the multi-national VIP-1 cohort comprising patients treated in different healthcare systems with diverging philosophies and financial resources and our more homogeneous German sub-cohort is most likely caused by the above mentioned inhomogeneity of the multi-national cohort. Thus, in contrast to the analysis of the multi-national VIP-1 cohort, our analysis among very old patients admitted to German ICUs showed that in octogenarians and nonagenarians not chronologic age but the bioenergetics state, stratified by the CFS, is a strong predictor of mortality.

The strengths of our study include its prospective multicentre character and the relatively high number of included patients. Time dependent confounders were minimized by limiting recruiting time to 8 months, thus time dependent confounders could be minimalized.

However, this study has limitations. No data on the proportion of included patients in relation to all ICU admissions nor data on patients that were not admitted to ICU due to triage decisions were recorded. For the sake of feasibility, neither long-term survival nor the functional status of the patients after ICU discharge were assessed. The used 9-point frailty scale is subjective with potentially high inter-rater variability. Nonetheless, in the context of acute ICU admission of vulnerable patients it is likely to be more feasible and easier to access than more objective scoring systems.

## Conclusion

In conclusion, the CFS is an easy determinable valuable tool for the prediction of mortality in very old patients, thus it can facilitate decision making for intensive care givers in Germany.

## Additional file

Additional file 1: Ethics Committees. (DOCX 111 kb)

## Abbreviations

APACHE: Acute Physiology and Chronic Health Evaluation (3 aspects: age points + acute physiology score + chronic health score)
CFS: Clinical Frailty Scale (from 1 to 9; 1 = very fit / 5 = mildly frail / 9 = terminally ill)
ICU: Intensive Care Unit
SAPS: Simplified Acute Physiology Score
SOFA: Sequential Organ Failure Assessment Score
VIP-1: The Very Old Intensive Care Patient Trial 1
