# Supplementary material for: Clinical Frailty Scale (CFS) reliably stratifies octogenarians in German ICUs: a multicentre prospective cohort study
Source: BMC Geriatr. 2018 Jul 13;18:162. doi: 10.1186/s12877-018-0847-7 (PMC6044022; doi:10.1186/s12877-018-0847-7)
Supplement: Supplementary file 1 — Ethics Committees. (DOCX 111 kb) [file 12877_2018_847_MOESM1_ESM.docx]

## **Ethics Committees**

Ethical approval was obtained from each study side with Duesseldorf (Ethic Committee of Medical Faculty of Heinrich-Heine University Duesseldorf, Germany; Head: Prof. Dr. T. Hohlfeld; study-number: 5644R and registration-ID: 2016075289 approved on 16^th^ September 2016) being the national headquarter for all german ethic committees. In the following you will find the names all involved ethic committees and the involved authors in brackets:

1. Ethic Committee of Medical Faculty of Heinrich-Heine University Duesseldorf, Germany (for JMM, AMN, MM, MK, and CJ).
2. Ethic Committee of Medical Faculty of the University Leipzig, Leipzig, Germany (for PS)
3. Ethic Committee of Medical Faculty Heidelberg, Heidelberg, Germany (for TB)
4. Ethic Committee of The Medical School of University Clinic Jena, Jena, Germany (for MF and FB)
5. Ethic Committee of Albert-Ludwigs-University Freiburg; Freiburg im Breisgau, Germany (for SU)
6. Ethic Committee of Charité Medical University Berlin, Berlin, Germany (for ALS and AL)
7. Ethic Committee of The Medical Council Thüringen, Erfurt Germany (for HE)
8. Ethic Committee of The Technical University Munich rechts der Isar, Munich, Germany (for SJS, KF, and CR)
9. Ethic Committee of The Medical Council Niedersachsen, Hannover, Germany (for TD)
10. Ethic Committee of The Medical Council Hessen, Limburg an der Lahn, Germany (for SS)
11. Clinical Ethic Committee of The University Clinic Essen, Essen, Germany (for RAJ)
12. Ethic Commission of Medical Faculty of the Goethe-University Frankfurt, Frankfurt am Main, Germany (for PM)
13. Ethic Committee of Medical Faculty of the University Bochum, Bochum, Germany (for TR)
14. Ethic Commission of Ulm Medical University, Ulm, Germany (for EB)
15. Ethic Commission of The Medical Council Rheinland-Pfalz, Mainz, Germany (for MS)
16. Ethic Commission of The University zu Lübeck, Lübeck, Germany (for TG)
